# Whole-genome resequencing reveals the uniqueness of Subei yak

**DOI:** 10.1093/jas/skae152

**Published:** 2024-06-04

**Authors:** Shaoke Guo, Tianjun Yu, Xingdong Wang, Shuangquan Zhao, Erjun Zhao, Ainierlitu, Teer Ba, Manyu Gan, Cunmei Dong, Naerlima, Lian Yin, Xikou Ke, Dawuti Dana, Xian Guo

**Affiliations:** Key Laboratory of Yak Breeding Engineering in Gansu Province, Lanzhou Institute of Husbandry and Pharmaceutical Sciences, Chinese Academy of Agricultural Sciences, Lanzhou 730050, China; Key Laboratory of Animal Genetics and Breeding on Tibetan Plateau, Ministry of Agriculture and Rural Affairs, Lanzhou, 730050, China; Center of Animal Husbandry and Veterinary Technology Services in Subei Mongolian Autonomous County of Gansu Province, Subei, 736300, China; Key Laboratory of Yak Breeding Engineering in Gansu Province, Lanzhou Institute of Husbandry and Pharmaceutical Sciences, Chinese Academy of Agricultural Sciences, Lanzhou 730050, China; Key Laboratory of Animal Genetics and Breeding on Tibetan Plateau, Ministry of Agriculture and Rural Affairs, Lanzhou, 730050, China; Center of Animal Husbandry and Veterinary Technology Services in Subei Mongolian Autonomous County of Gansu Province, Subei, 736300, China; Center of Animal Husbandry and Veterinary Technology Services in Subei Mongolian Autonomous County of Gansu Province, Subei, 736300, China; Center of Animal Husbandry and Veterinary Technology Services in Subei Mongolian Autonomous County of Gansu Province, Subei, 736300, China; Center of Animal Husbandry and Veterinary Technology Services in Subei Mongolian Autonomous County of Gansu Province, Subei, 736300, China; Center of Animal Husbandry and Veterinary Technology Services in Subei Mongolian Autonomous County of Gansu Province, Subei, 736300, China; Center of Animal Husbandry and Veterinary Technology Services in Subei Mongolian Autonomous County of Gansu Province, Subei, 736300, China; Center of Animal Husbandry and Veterinary Technology Services in Subei Mongolian Autonomous County of Gansu Province, Subei, 736300, China; Center of Animal Husbandry and Veterinary Technology Services in Subei Mongolian Autonomous County of Gansu Province, Subei, 736300, China; Center of Animal Husbandry and Veterinary Technology Services in Subei Mongolian Autonomous County of Gansu Province, Subei, 736300, China; Center of Animal Husbandry and Veterinary Technology Services in Subei Mongolian Autonomous County of Gansu Province, Subei, 736300, China; Key Laboratory of Yak Breeding Engineering in Gansu Province, Lanzhou Institute of Husbandry and Pharmaceutical Sciences, Chinese Academy of Agricultural Sciences, Lanzhou 730050, China; Key Laboratory of Animal Genetics and Breeding on Tibetan Plateau, Ministry of Agriculture and Rural Affairs, Lanzhou, 730050, China

**Keywords:** population structure, SNP, Subei yak, whole-genome resequencing

## Abstract

Subei yak is an essential local yak in the Gansu Province, which genetic resource has recently been discovered. It is a meat-milk dual-purpose variety with high fecundity and relatively stable population genetic structure. However, its population genetic structure and genetic diversity are yet to be reported. Therefore, this study aimed to identify molecular markers of Subei yak genome by whole-genome resequencing, and to analyze the population structure and genetic diversity of Subei yak. This study screened 12,079,496 single nucleotide polymorphism (SNP) molecular markers in the 20 Subei yaks genome using whole-genome resequencing technology. Of these SNPs, 32.09% were located in the intronic region of the genome. Principal component analysis, phylogenetic analysis, and population structure analysis revealed that the Subei yak belonged to an independent group in the domestic yak population. A selective clearance analysis was carried out on Subei yak and other domestic yaks, and the genes under positive selection were annotated. The functional enrichment analysis showed that Subei yak possessed prominent selection characteristics in terms of external environment perception, hypoxia adaptation, and muscle development. Furthermore, Subei yak showed excellent muscle fat deposition and meat quality traits. Thus, this study will serve as a reference for discovering population structure, genetic evolution, and other unique traits of Subei yak and for expanding the genetic variation catalog of yaks.

## Teaser

The whole-genome resequencing data analysis showed that the Subei yak belonged to an independent group in the domestic yak population.

## Introduction

Yak (*Bos grunniens*) is a unique species living in the high-altitude areas of the Qinghai Tibet Plateau and its surrounding areas, such as Qinghai, Tibet, Sichuan, and Gansu province (Ma et al., 2013). Yak is known to provide people with meat, milk, plush, and labor, which have irreplaceable ecological, social, and economic importance in alpine pastoral areas (Qiu et al., 2012, 2015). Yak has been domesticated from the original wild yak (*Bos mutus*) population for thousands of years. Due to the difference in natural environment, yak genetic resources with different ecological adaptability and characteristics have been gradually formed. A developed breed is a kind of livestock breed formed by artificial selection and cultivation in a planned and purposeful way under good breeding conditions. Currently, there are 18 local and two developed breeds of yak in China.

With the rapid development of molecular genetics and sequencing technology, molecular markers have been widely used to understand the origin and evolution of animals. In domestic animals, genetic markers, such as single nucleotide polymorphism (SNP), insertion or deletion (indel, < 50 bp), and copy number variation have been shown to result in the most extensive genomic variations (Chen et al., 2009; Zhang et al., 2016; Serres-Armero et al., 2017). Furthermore, SNP molecular markers are characterized by wide distribution, high density, abundant loci, and high genetic stability in the genome. Currently, they are the most commonly used means to evaluate the genetic diversity in animal populations. Several studies have reported the identification of SNP molecular markers in various non-model organisms. Additionally, SNPs are applied in genetic map construction, species identification, biological population analysis, genetic structure, and phylogenetic analysis (Drywa et al., 2014; Wenne et al., 2016; Nugent et al., 2017; Förster et al., 2018; Wang et al., 2018; Sabahat et al., 2020). Multiple studies have investigated the domestication process and population structure of the yak. Qiu et al. (Qiu et al., 2015) performed whole-genome sequencing and identified SNPs in the genome of wild and domestic yak, tracing back to 7,300 yr ago, when the domestication of yaks started. The authors also used population genetic research methods to identify some selected genes related to yak domestication phenotype, such as the *ADGYAP1R1* gene, which was related to fear response. Harmful variation is also called nonsynonymous mutation, including missense mutation and nonsense mutation. Harmful mutations usually have a negative impact on the health of organisms and may reduce their adaptability. Xie et al. (Xie et al., 2018) used genome resequencing data to evaluate the pattern of genome-wide harmful variation in domestic and wild yak, and revealed a significant increase in the number of harmful SNPs of domesticated yaks. Deleterious genes were found to be associated with the perception of smell and detection of chemical stimulus (Xie et al., 2018). Thirty-six genes associated with Mendelian genetic diseases were identified, involving sensory perception, bone development, and the nervous and immune systems. Zhang et al. (Zhang et al., 2021) compared the structural variants of wild and domestic yak via long-read whole-genome sequencing and screened the possible target genes artificially selected in the process of yak domestication. Wu et al. (Wu et al., 2018) used population genetic analysis methods to discover that genes under domestication selection in cattle (for example, *MITF*) were introgressed from domestic cattle to yak. Meanwhile, gene haplotypes related to plateau adaptation were passed from yaks to plateau yellow cattle through introgression (Wu et al., 2018). Despite this, yaks are currently facing a severe test of population degradation, and hence there is an urgent need to improve and cultivate new varieties.

**Figure 1.**
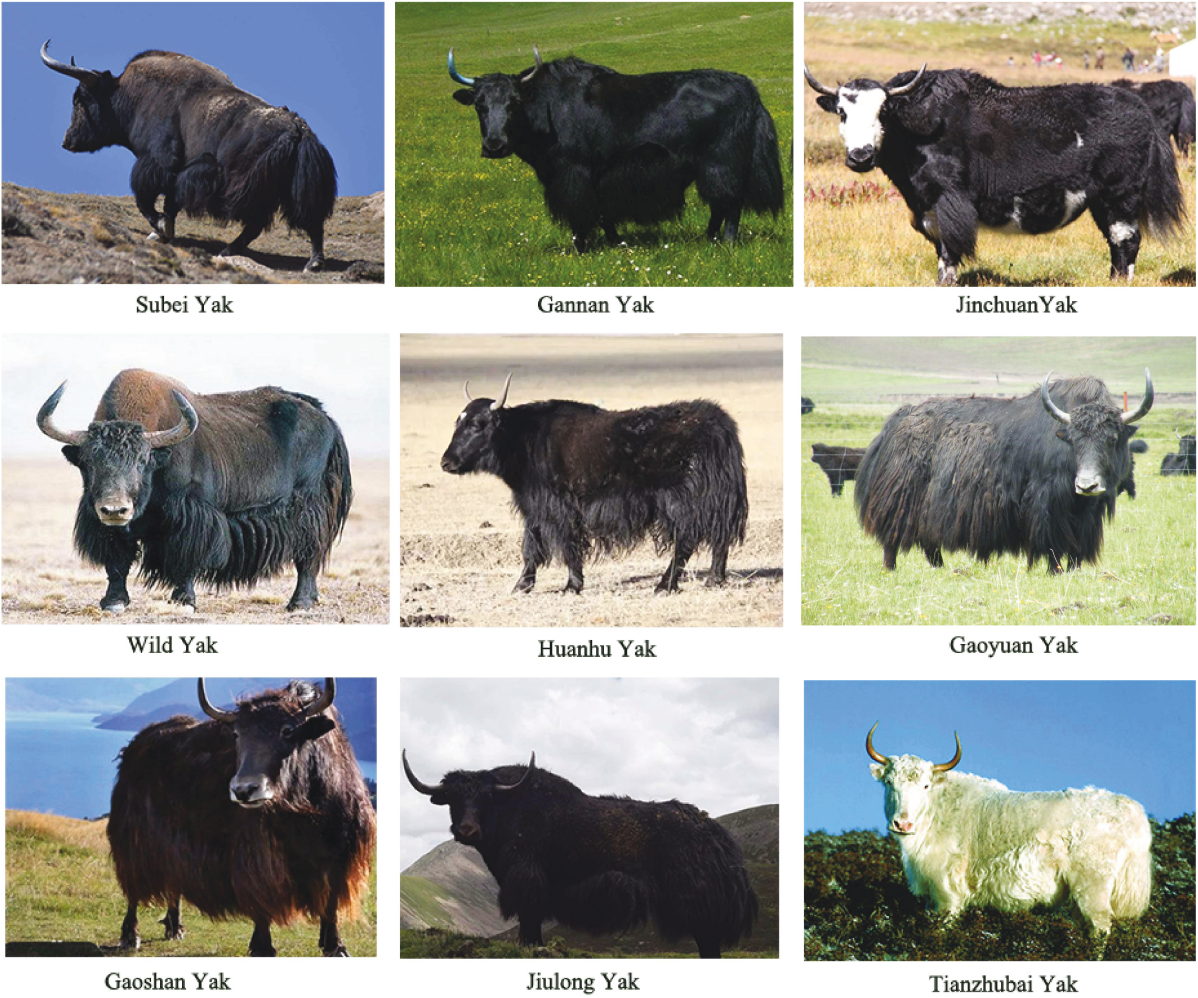
The appearance characteristics of Subei yak and other yaks.

Gansu Province is located in the northwest of China, located in the middle and upper reaches of the Yellow River. It is the intersection of the three plateaus of the Loess Plateau, the Qinghai-Tibet Plateau, and the Inner Mongolia Plateau. Subei yak is a unique yak breed recently discovered in the Gansu Province. Subei yak is primarily distributed in the alpine areas at about 3,800 m altitude. The core production area of the livestock is located in Yanchiwan Township, Subei Mongolian Autonomous County, Gansu Province. Subei yak are robust, and their fur is black in most cases, which is closer to the body shape of wild yak (Figure 1). They primarily produce meat and milk. Subei yak meat is rich in high-quality protein and fatty acids, and the contents of lysine, isoleucine, and glutamic acid in meat are higher than those of Simmental beef and Gansu Tianzhu yellow beef (Ding et al., 2023a). The milk density and protein content of Subei yak colostrum were reported to be significantly higher than those of Huanhu yak and Gannan yak (Ding et al., 2023b). The Subei yak population has stable genetic performance and high fecundity. As a newly discovered and vital local yak genetic resource, the population structure, genetic characteristics, and unique traits of Subei yak have not been studied yet.

In this study, the SNP molecular markers of Subei yak were detected by whole-genome resequencing technology to understand the population structure, genetic diversity, and genetic relationship of Subei yak. Furthermore, the population structure and genetic distance of Subei yak combined with the resequencing data of other domestic and wild yaks were analyzed. Thus, this study analyzed the germplasm characteristics and population structure of Subei yak and provided molecular markers and a theoretical basis for the conservation and utilization of Subei yak and variety identification.

## Materials and Methods

### Ethics statement

All animal procedures were performed according to the China Council on Animal Care guidelines and the Ministry of Agriculture of the People’s Republic of China. The yak handling procedures were approved by the Animal Care and Use Committee of the Lanzhou Institute of Husbandry and Pharmaceutical Sciences Chinese Academy of Agricultural Sciences (Permit No: SYXK-2014-0002).

### Sample collection

**Figure 2.**
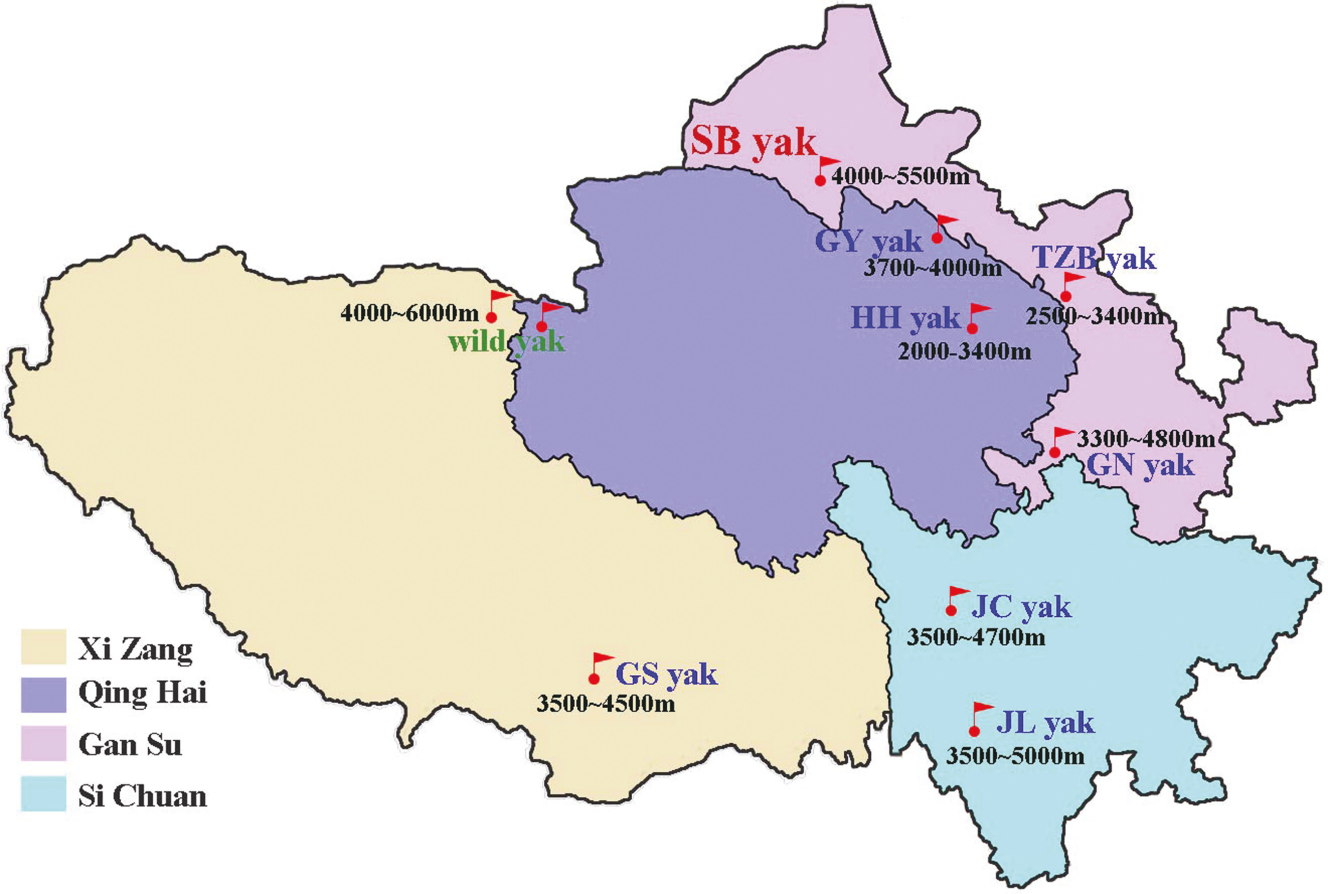
The geographical distribution of yaks selected in this study.

Twenty 4-yr-old Subei yaks (SB-YAK) were selected from Yanchiwan Township, Subei Mongolian Autonomous County, Gansu Province, China (38° 98″ N, 96° 06″ E). Forty-seven different domestic yak (DO-YAK) aged 4 to 6 years old in Gansu, Qinghai, Tibet, and Sichuan provinces were selected (Figure 2), including Tianzhu white yak (TZB-YAK, *n* = 10), Gannan yak (GN-YAK, *n* = 7), Huanhu yak (HH-YAK, *n* = 4), Gaoyuan yak (GY-YAK, *n* = 3), Gaoshan yak (GS-YAK, *n* = 4), Jinchuan yak (JC-YAK, *n* = 9), and Jiulong yak (JL-YAK, *n* = 10). Blood samples were collected from the jugular vein, anticoagulated with EDTA, and stored at −80 °C for genomic DNA extraction. All animal procedures were performed according to the China Council on Animal Care guidelines and the Ministry of Agriculture of the People’s Republic of China.

### DNA extraction and library construction

Genomic DNA was extracted from yak blood samples using TIANamp Blood DNA Kit (TIANGEN, Beijing, China). DNA sequencing libraries were constructed after gel electrophoresis verified the quality and length of DNA of the samples. OE Biotech Co., Ltd. (Shanghai, China) performed the sequencing and analysis. The library was constructed with a TruSeq Nano DNA LT Sample Preparation Kit (Illumina, San Diego, CA, USA). The S220-focused ultrasound was used to fragment the genomic DNA into 350 bp (Covaris, USA). Adapters were ligated to the 3ʹ end of the sheared fragments. Post-PCR amplification and purification, the final libraries were sequenced on the Illumina sequencing platform HiSeq X Ten platform (Illumina Inc., San Diego, CA, USA), and 150 bp paired-end reads were generated.

### Quality control and reference genome alignment

The obtained raw reads were filtered by fastp software (Chen et al., 2018; version: 0.20.0). The filtering process included the following: (1) removal of linker sequences, (2) removal of reads with N (i.e., non AGCT bases) ≥ 5, (3) removal of the average base mass value < 20, (4) removal of the base sequencing error rate ≥ 1 % and Phred score < Q20, and (5) Steps 1 to 3 were followed by, the removal of reads with a length < 75 bp or an average base mass value < 15, to obtain clean and high-quality reads. The clean reads were aligned to the reference genome (version: LU_Bosgru_v3.0) using BWA (Bolger et al., 2014; Version: 0.7.12). The algorithm used for alignment was bwa mem, and the default parameters were used. The SAMtools package was used to format and sort the aligned results (Li et al., 2009; Version: 1.9), and the duplicate reads were marked with MarkDuplicates using Picard (Version: 2.18.17) software. The results were compared with the Qualimap software (Okonechnikov et al., 2016) for statistics.

### Detection and annotation of population Snps and indels

The Haplotypecaller module of GATK (Version: 3.8.1; McKenna et al., 2010) software was used to detect SNPs. The filtering parameters for GATK are QD < 2.0 / MQ < 40.0 / Fisher strand > 60.0 / SOR > 3.0 / MQRankSum < −12.5 / ReadPosRanSum < −8.0. QualByDepth (QD): the credibility of the mutation site divided by the number of unfiltered nonreference reads. RMSMappingQuality (MQ): the square root of the comparison quality in all samples. Fisher strand: Fisher’s exact test evaluates the likelihood of the current mutation being a strand bias. StrandOddsRatio (SOR): a comprehensive evaluation of the likelihood of Strand bias. MappingQualityRankSumTest (MQRankSum): evaluate credibility based on the comparison quality of reads between REF and ALT. ReadPosRankSumTest (ReadPosRankSum): evaluate the credibility of mutations by their position in the read. Individuals with more than 20% missing genotypes and sites with more than two different genotypes were removed. Polymorphic sites were also filtered to retain SNPs with a minor allele frequency > 5%. All the variants were annotated with SnpEff (Version: 4.3T; Cingolani et al., 2012). The SNP mutation data of all samples was combined using GATK software.

### Group hierarchical analysis

In order to make SNP inference of population stratification more accurate, the entire genome’s SNPs were linkage-disequilibrium pruned using PLINK software (parameter:—indep-pairwise 50 5 0.5; Purcell et al., 2007), and SNPs without tight linkage were selected for subsequent phylogenetic tree analysis, principal component analysis, and population structure analysis. The phylogenic tree was constructed by the Neighbor-joining (NJ) method (Bo et al., 2015). The IBS matrix was calculated by PLINK, and the neighbors in PHYLIP (Felsenstein, 1989) were used to construct the phylogenetic trees for all samples. The eigenvectors were calculated via the EIGENSOFT (Price et al., 2006) software, and the first four principal components were extracted and plotted. The ADMIXTURE (Alexander et al., 2009) software was used to analyze the population structure according to K = 2 to K = 4. Ten different seeds were selected for 10 repeated analyses, and pong (Behr et al., 2016) was used to cluster the results 10 times according to the cross-validation error to determine the optimal K value.

### Selective clearance analysis

Except for population stratification analysis, all other analyses (including selective clearance analysis) were conducted using snp loci before LD pruning. The population fixation statistics (FST) and nucleotide diversity (θπ) for each sliding window (in 100 kb windows with 10 kb step size) were calculated using VCFtools to identify the genomic regions under selection (Danecek et al., 2011). The putative selection targets were designated as the top 5% log-odds ratios for both θπ and FST. Gene Ontology (GO), and Kyoto Encyclopedia of Genes and Genomes (KEGG) functional annotations were performed on the genes in the selected regions of each group, followed by the corresponding enrichment analysis. The number of genes included in each GO entry or KEGG pathway entry was counted, and the significance of the enrichment of selected genes was calculated in each GO entry or KEGG pathway entry by the hypergeometric distribution test.

## Results

### SNP calling and distribution

In this study, 638.97G raw reads were obtained from sequencing 20 Subei yak individuals (Supplementary Table S1). The mean GC content was 42.61%, and the GC distribution is shown in Supplementary Figure 1A. As can be seen in the figure the N content is low in the reads. 635.09G clean reads were obtained after quality filtering. On mapping the clean reads to the reference genome (LU_Bosgru_v3.0), the average mapping rate of Subei yak samples was determined to be 98.65%. The average sequencing depth was 10.47×. On average, 95.49% (SD: 0.003) of the genome is covered at 1X depth, while an average of 46.99% (SD: 0.113) is still covered at 5X depth (Supplementary Table S2). The sequencing depth of other domestic yak breeds (a total of 47 yaks) was 10×. The sequencing data of 10 wild yaks from the European Nucleotide Archive (EMBL-EBI) was downloaded under accession code PRJNA285,834 (Qiu et al., 2015) for subsequent analysis, with an average sequencing depth of 6.7×.

**Figure 3.**
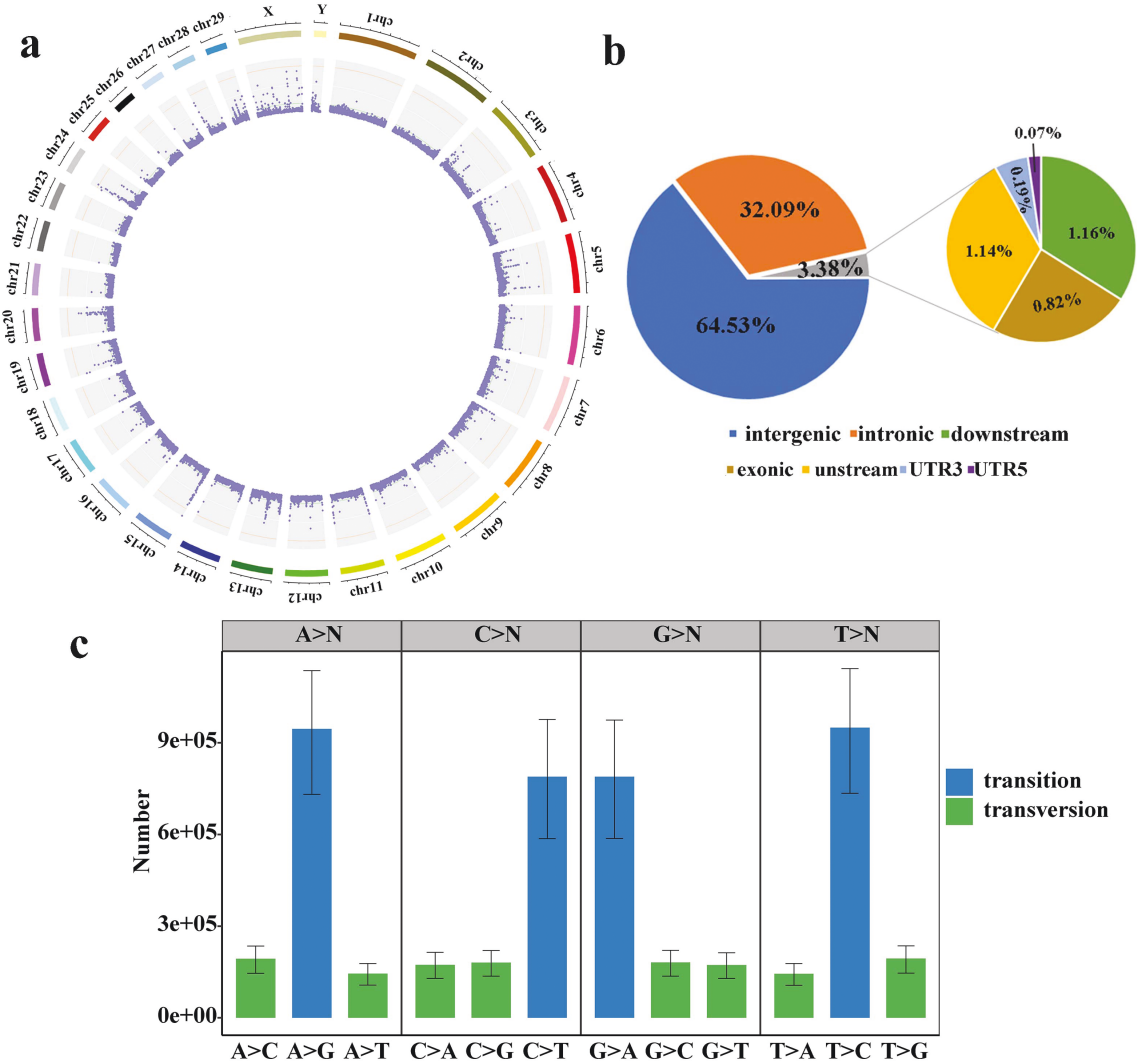
Summary of single nucleotide polymorphism (SNP) statistical information in Subei yak. (A) The distribution of SNP is detected on each chromosome. (B) Statistics of SNP annotation types. (C) The statistics of transition and translation types in SNP.

A total of 12,079,496 SNP loci were obtained in 20 individuals after filtering and screening the population SNP data. Statistical analysis of SNP variations on various chromosomes (Supplementary Table S3), 718,630 SNPs were observed on chromosome 1 (Figure 3A), which was the chromosome with the highest number of SNPs. Analysis of the location of SNP in gene regions revealed that 64.53% of the SNPs were located in the intergenic region, 32.09% were located in the intronic region, 1.14% and 1.16% of the SNPs were located in the upstream and downstream regions, respectively. Only 0.82% were located in the exonic region (Figure 3B). There were 50,241 synonymous SNPs and 48,095 nonsynonymous SNPs, 1,015 stop gain SNPs, and 145 stop loss SNPs in the exonic region. The ratio of transition mutations (75,944,050) to transversion mutations (30,412,677) was 2.50, and the most common substitution mutations were T > C and A > G (Figure 3C).

### Population stratification analysis

In order to understand the population structure and genetic diversity of Subei yak, population stratification was analyzed via three levels: PCA analysis, phylogenetic tree, and population structure. Of 2,619,781 SNPs without close linkage were selected for population stratification analysis with SNPs of wild yaks and other domestic yaks.

**Figure 4.**
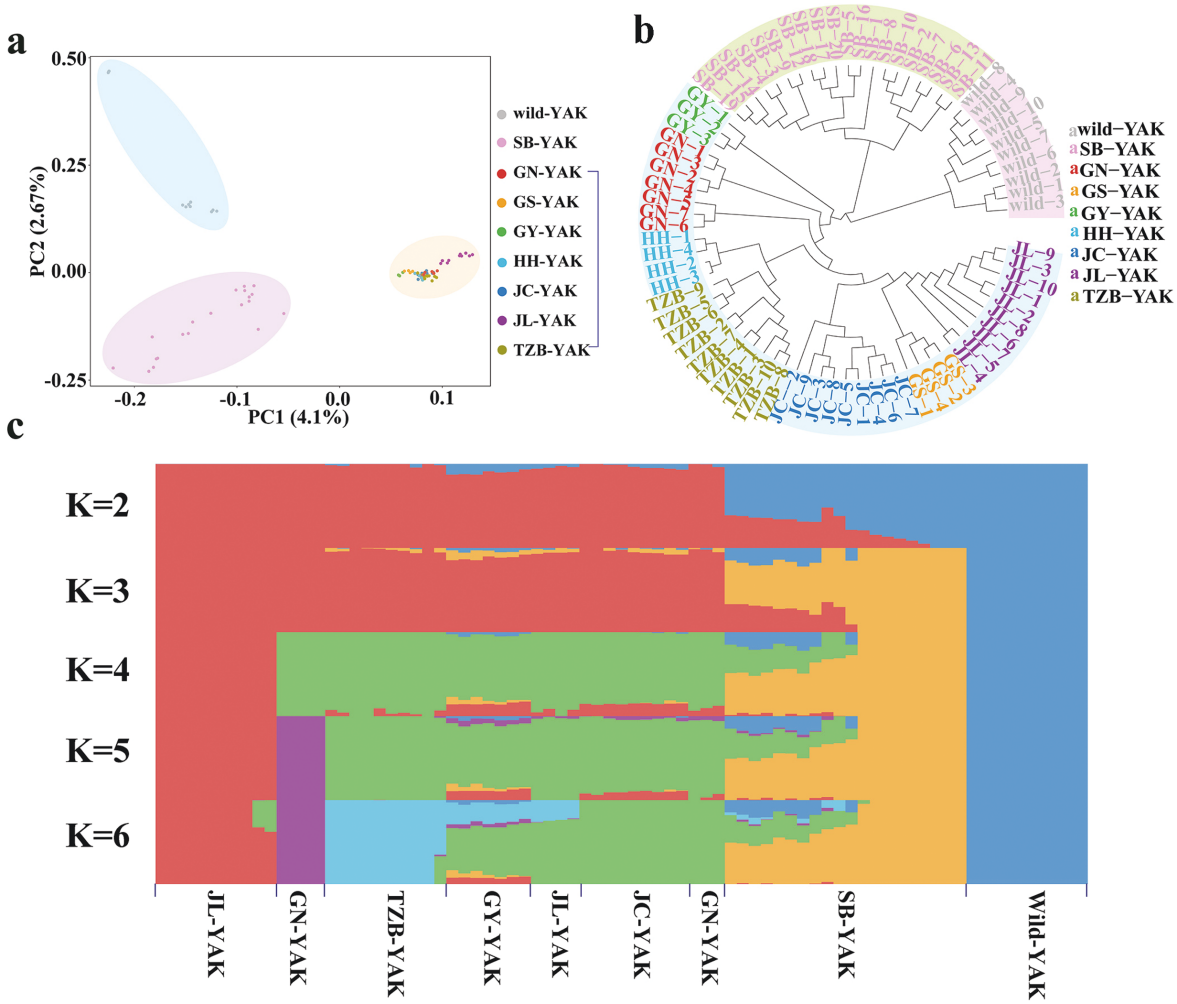
Population stratification analysis of Subei yak and other yaks. (A) The principal component analysis of Subei yak and other yaks. (B) The phylogenetic analysis of Subei yak and other yaks. (C) The population structure analysis of Subei yak and other yaks. Each column of the X-axis represents a yak sample, and each row of the Y-axis represents the corresponding value of K. Subei yak (SB-YAK), Tianzhu white yak (TZB-YAK), Gannan yak (GN-YAK), Huanhu yak (HH-YAK), Gaoyuan yak (GY-YAK), Gaoshan yak (GS-YAK), Jinchuan yak (JC-YAK), and Jiulong yak (JL-YAK).

Figure 4A shows the results of the PCA analysis. In the 2D space composed of the two primary characteristics obtained from PCA analysis, Subei yak, wild yak, and other domestic yak were divided into three clusters with no overlap. Next, the NJ method was used to construct a phylogenetic tree for all yak individuals (Figure 4B). The yak population was clustered into two independent branches on the phylogenetic tree: one wild yak and one domestic yak. In the branch of domesticated yak, Subei yak was separately divided into a small branch, while other domesticated yaks were divided into another small branch, consistent with the results of principal component analysis. ADMIXTURE software was used to analyze the population structure (K = 2 ~ K = 6). Population structure analysis showed that ten different seeds were selected for 10 repeated analyses, and the results were clustered 10 times. The detailed results are shown in Supplementary Table S4. The optimal K was determined according to the cross-validation error. The optimal K of 77 samples was 2 from the results of 10 repetitions (Supplementary Fig 1b). As shown in Figure 4C, when K = 2, the yak population was divided into two groups: wild yak and domestic yak. Some individuals in these two populations had genetic components of another population; When K = 3, the yak population was divided into three groups: wild yak, Subei yak, and other domestic yaks. Subei yak population contained both domestic and wild yak population genetic components. In general, Subei yak are far from wild yak and other domestic yaks, and there exists some differentiation among their population.

### Genetic differentiation index and genetic diversity

The genetic differentiation analysis based on the resequencing data showed that the genetic differentiation index Fst value of Subei yak and wild yak was 0.03292, and that of Subei yak and other domestic yak was 0.03434. The 0 < Fst < 0.05 indicated random mating between the two populations, the level of genetic differentiation was estimated to be negligible, and no isolation exists. There is also frequent gene flow between the two populations. These results indicate that the genetic differentiation among the yak populations could be ignored. Genetic diversity is usually represented by nucleotide diversity (pi). The higher the genetic diversity (θπ value), the higher the degree of genetic variation in the population. The analysis of the resequencing data showed that the pi values of wild yak, domestic yak, and Subei yak were 0.00117, 0.00128, and 0.00131, respectively, indicating the absence of significant difference between their genetic diversity.

### Selective clearance analysis

**Figure 5.**
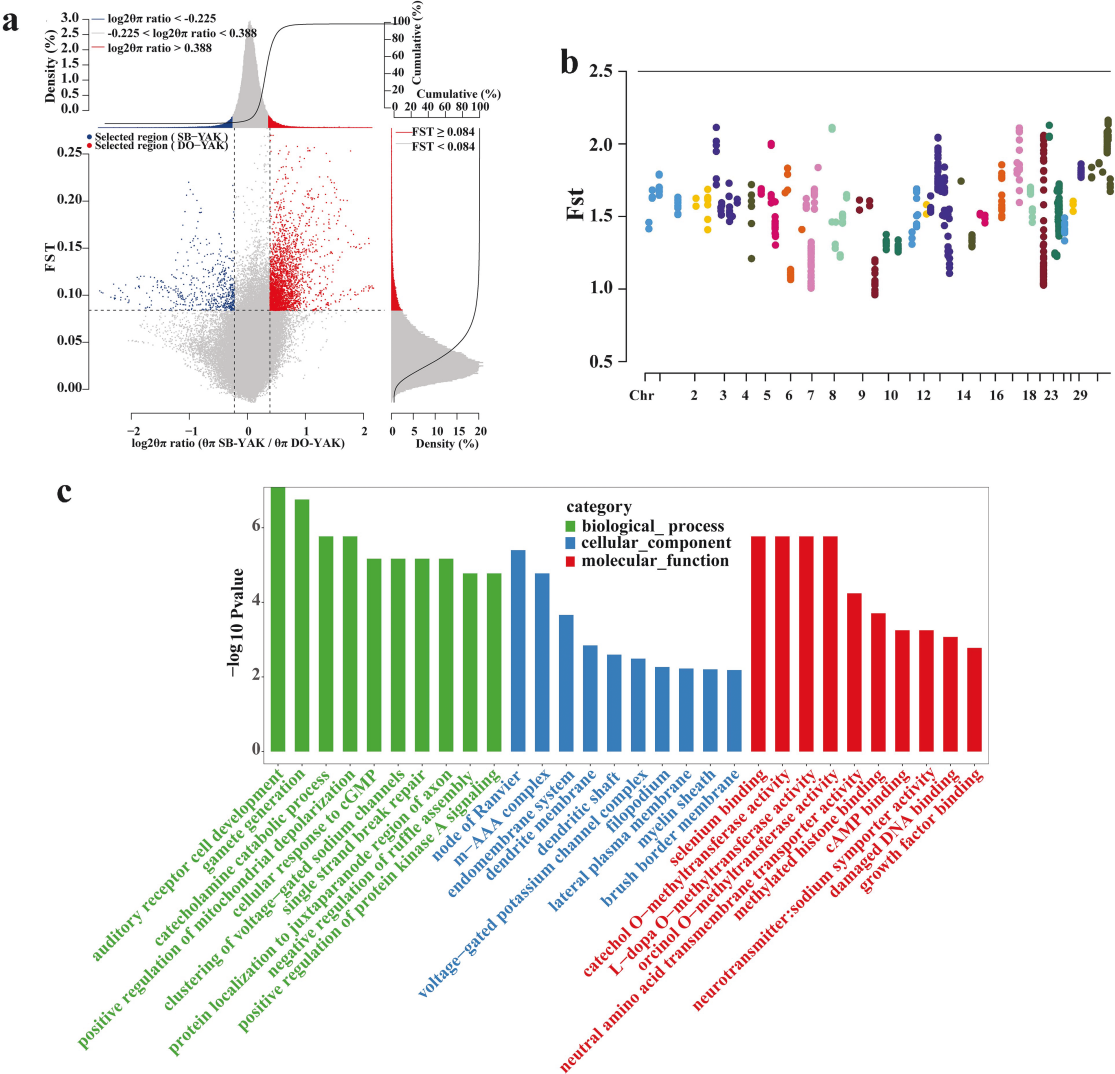
Results of location, functional annotation, and gene ontology (GO) analysis of Subei yak in selected areas. (A) The Location map of selected areas among Subei yak compared with other domestic yaks. The top 5% area of Fst value and the ratio of θπ is selected as the selected area, with a window of 200 kb and a step size of 20 kb. The data points in the left area represent the selected area of Subei yak. (B) Manhattan plots of genome-wide distribution of Fst values for comparing Subei yak and other domestic yaks. (C) GO enrichment results of selected region genes in Subei yak.

**Figure 6.**
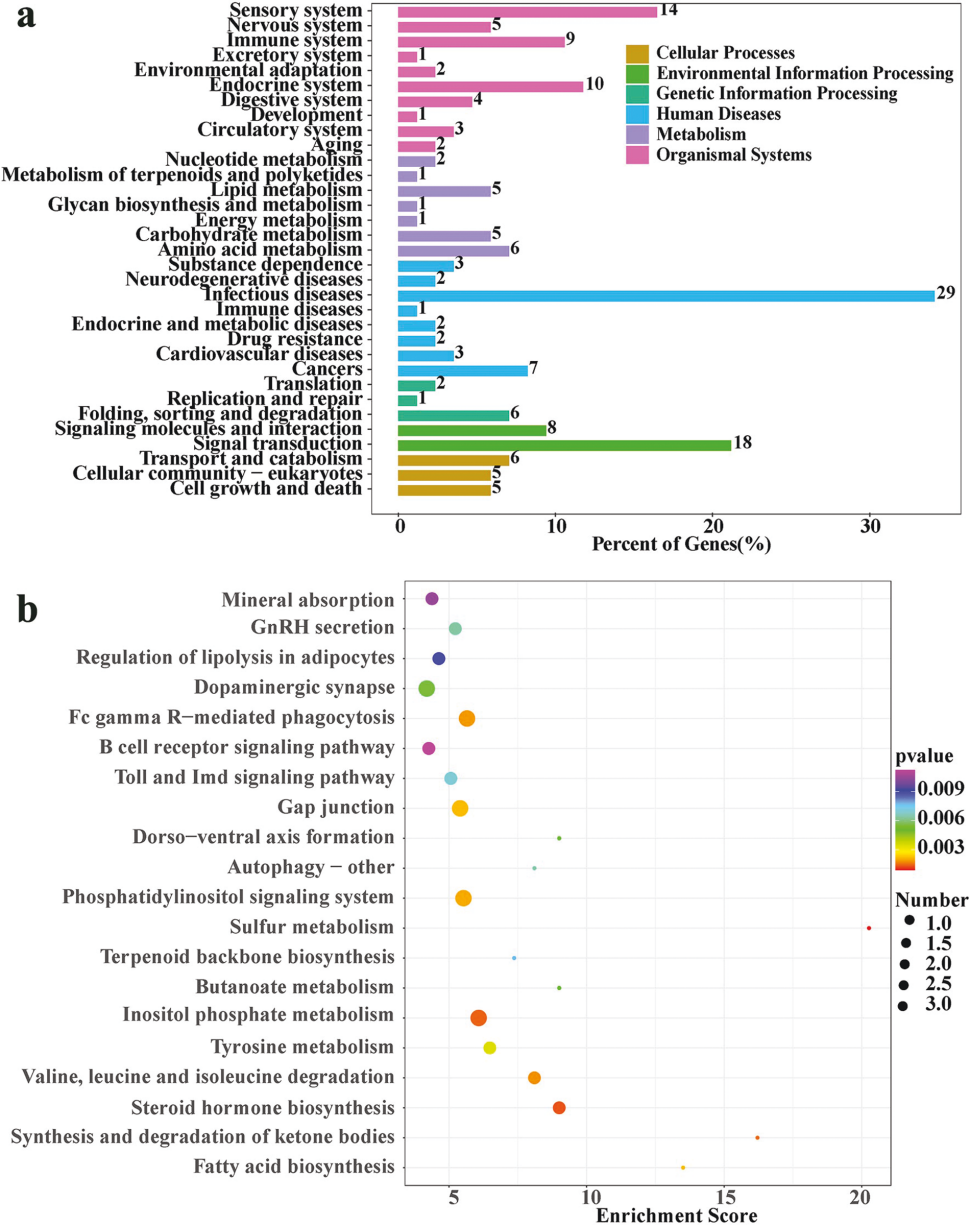
Kyoto Encyclopedia of Genes and Genomes (KEGG) enrichment analysis of Subei yak in selected areas. (A) KEGG classification annotation results of selected region genes in Subei yak. The numbers in the figure refer to the number of genes enriched in this pathway. (B) KEGG pathway enrichment results of selected region genes in Subei yak. Enrichment score = (ListHits / ListTotal) / (PopHits / PopTotal). The size of the circle in the figure represents the number of genes enriched in this pathway, and the color of the circle represents the size of the *P*-value.

The selected regions of Subei yak and other domestic yak genomes were screened by the θπ ratio and the FST values. The top 5% of the area is extracted and selected. The results are shown in Figure 5A. log2 θπ ratio < −0.225 and FST ≥ 0.084 were selected to be the parameters of positive selection of Subei yak (Supplementary Table S5). Figure 5B shows the distribution of the Fst value of the positive selection signal in Subei yak on the whole genome. Through annotation, it was found that these SNPs fell in the relevant regions of 372 coding genes (Supplementary Table S6). The functional enrichment of these positive selection genes revealed that their functions were primarily related to the traits of domestication syndrome. Furthermore, GO analysis revealed that these genes were significantly enriched in biological processes, such as auditory receptor cell development, gamete generation, and positive regulation of mitochondrial depolarization (Supplementary Table S7; Figure 5C). They were also significantly related to methyltransferase activity, cAMP binding, damaged DNA binding, and growth factor binding (Figure 5C). Furthermore, KEGG classification results demonstrated that the selected genes were related to the domestication characteristics and some economic traits of the yaks (Supplementary Table S8), which are mainly divided into cell growth and death, transport and catabolism and other cellular processes; some processes related to environmental information processing and genetic information processing; metabolic processes such as carbohydrate metabolism, energy metabolism, and lipid metabolism; endocrine system, environmental adaptation, immune system, neutral system, sensor system, and other related organic systems (Figure 6A). Supplementary Table S9 shows the detailed results of KEGG pathway enrichment analysis. The top 5 KEGG predicted pathways include sulfur metabolism, steroid hormone biosynthesis, inositol phosphate metabolism, synthesis and degradation of ketone bodies, valine, leucine, and isoleucine degradation (Figure 6B). Functional enrichment analysis indicated that the selected genes were involved in the growth, muscle development, reproduction, and other essential life processes of Subei yak.

## Discussion

In the long process of evolution, yaks have secured stable genetics, unique mechanisms in morphology, physiology, reproductive performance (Prakash et al., 2008; Krishnan et al., 2016, 2018), and other aspects through harsh natural selection and formed distinctive yak genetic resources. The Subei yak is a newly discovered yak population in the Gansu Province. Subei yak meat is tender, and its milk is of high quality. The population structure of the yak is stable, and its reproductive performance is strong compared with other domestic yaks. For these reasons, the Subei Yak is popular and has high production value. In order to understand the genetic structure of Subei yak, its evolution, and its kinship with other yak breeds, the SNP loci in its genome were sequenced, and the population structure combined with other yak data was analyzed. The genetic diversity of Subei yak was studied, and the uniqueness of Subei yak different from other yak breeds was evaluated. In this study, 12.07 million SNPs were obtained from the genomes of 20 Subei yaks through high-throughput sequencing. The sequencing results revealed many breed-specific variant sites, significantly expanding the public SNP database of Chinese yaks.

A PCA analysis revealed that genetically speaking, Subei yaks, domestic yaks in other regions, and wild yaks can be divided into three distinct groups. In this study, the SNP loci were used to construct the evolutionary tree, Because the number of loci was too large, the calculation was increased, and these yak individuals of different breeds also had close kinship. Hence, the NJ method was used with the highest computational efficiency to construct the tree. Phylogenetic analysis shows that all yaks are divided into two branches: wild yaks and domesticated yaks. Lan et al. (2018) conducted a phylogenetic analysis on Jinchuan yak, wild yak, and other domesticated yaks. The evolutionary tree results indicate that the yak population in China is mainly divided into wild yaks and domesticated yaks. Jinchuan yak is an independent small branch of yak in China, which is very different from other breeds of yak. When we added the Subei yak to the phylogenetic analysis, it belonged to an independent small branch of the domesticated yak branch. Our results indicate that there are some differences in genetic evolution between Subei yak and other domesticated yaks.

Previous research by Qiu et al. (2015) and Li et al. (2023) has shown that when the population structure analysis K = 2, the population was divided into two subgroups: domestic yaks and wild yaks. This was consistent with our results. Chai et al. (2020) conducted a population structure analysis on 92 domestic and wild yaks from different regions, and found that when K = 2, the yak population can be divided into domestic yak and wild yak. However, when K = 3 to 5, the yak sample cannot be divided into different ancestors, indicating a wide range of genetic mixing among domestic yaks. In our results, when K = 3, Subei yak can be separated from the domestic yak subpopulation to form an independent subpopulation. Population structure analysis showed that Subei yak carried both the genetic components of domestic yak and wild yak. There is continuous gene flow between groups, although the morphological differences between Subei yak and other domestic yaks are minor. Some genetic splitting was observed between Subei yak and other domestic yaks. Meanwhile, the Fst values of Subei yak and domestic yak and Subei yak and wild yak are lower than 0.05, indicating gene exchange among the three yak groups. Specific differences exist between the Subei yak and other domestic yaks, indicating that the Subei yak has characteristics independent of the domestic yak. It was found in this study that despite the gene flow between Subei yak and other domestic yak, selection signals could still be detected at loci affecting morphology and behavior in Subei yak. GO enrichment analysis of the genes annotated at the sites of the selected region revealed that the selected genes were significantly enriched in biological processes related to the development of the nervous system. Subei yak have been living in high-altitude areas for a long time, and in this environment, they need to adjust their metabolism to maintain physical function. Perceiving these changes is crucial for regulating short-term and long-term physiological and behavioral responses. The auditory receptor relies on neurotransmission, receiving signals encoded in environmental noise, and regulates selective attention to sensory stimuli (Uthaiah and Hudspeth, 2010; Fuchs and Lauer, 2019). The genes that are positively selected in Subei yaks are significantly enriched in the process of auditing receiver cell development, which may be due to the sensitivity of Subei yaks to external environmental perception, making them better able to adapt to high-altitude and cold climates.

Meanwhile, genes that are positively selected in the Subei yak are significantly enriched in the process of positive regulation of mitochondrial depolarization. The mitochondrial depolarization process induces mitophagy that can effectively remove damaged mitochondria, playing an essential role in mitochondrial and metabolic homeostasis, energy supply, neuronal survival, and health (Li and Mu, 2017; Lou et al., 2020). Studies have reported that mitochondrial depolarization leads to the release of more reactive oxygen species from mitochondria (Zhong et al., 2018) that sharply promotes Ca^2+^ alternation through the redox effect of ROS, affecting the heart’s normal rhythm (Pandey et al., 2021). Wang et al. (Wang et al., 2016) showed that some differential gene expression exists related to the oxygen supply and defense against hypoxia in the gene expression profile of the heart between the yak in the high-altitude area and the cattle in the low-altitude area. This differential expression suggests that the Subei yak has a greater advantage in adapting to high-altitude and low-oxygen environments than other domestic yaks, which may be why they survive at high altitudes in the Qilian Mountains.

Early research has shown that active selection of brain and nerve-related genes and functional pathways is critical in the early stages of rabbit and cattle domestication (Carneiro et al., 2014; Montague et al., 2014). The genes that were positively selected in Subei yaks were significantly enriched in the cellular response to the cGMP process. CGMP regulates calcium homeostasis and light transduction in animals (Domek-Łopacińska and Strosznajder, 2005). It is also known to regulate catecholamine-dependent signal transduction in cardiomyocytes (Stangherlin et al., 2011). Catecholamines are neurotransmitters or hormones in higher animals and play an essential role in regulating mood, motivation, arousal, and plasticity and promoting metabolism (Macdonald et al., 1985; Tsunoda, 2008; Gasser, 2021). In addition, among the positive selection genes of Subei yak, we found Phosphodiesterase type 2A (PDE2A), which affects memory, learning, and cognition (Nakashima and Matsui, 2020). *PDE2A* is expressed in both the peripheral and central nervous systems, regulating intraneuronal cGMP and cAMP levels in regions of the brain related to mood, perception, attention, learning, and memory (Trabanco et al., 2016). Qiu et al. (Qiu et al., 2015) and Lan et al. (Lan et al., 2018) reported that genes related to neurodevelopment and behavior were involved in yak domestication. The GO analysis of this study confirmed that the genes related to neurodevelopment were positively selected in Subei yak, and *PDE2A* gene may play a key role in regulating nervous system development. In brief, the Subei yak showed prominent selection characteristics in perceiving the external environment, hypoxia adaptation, and behavior.

KEGG enrichment analysis revealed that the positively selected genes in Subei yak were significantly enriched in pathways related to muscle development, such as synthesis and degradation of ketone bodies, Inositol phosphate metabolism, and Fatty acid biosynthesis (Wei et al., 2022). Amino acids are essential taste-active components in meat and play a decisive role in meat flavor. Several potential selective scanning regions associated with domestication related to specific breed traits were also identified in Subei yak, such as the meat quality trait-related gene Acyl-CoA synthetase family member 3 (ACSF3). *ACSF3* is associated with fatty acid synthesis functions (Bernal Rubio et al., 2015; Bovo et al., 2015; Cai et al., 2020). He et al. (He et al., 2021) showed that the polymorphism of *ACSF3* can be used as a useful molecular marker for selection in the breeding of intramuscular fat deposition in beef cattle. This indicated that Subei yak showed excellent muscle fat deposition and meat quality traits. In addition, among the genes significantly enriched in the pathway, the study also found a gene 3-hydroxy-3-methylglutaryl-coenzyme A synthase 1 (HMGCS1) associated with lactation persistence (van Dorland et al., 2009), melanocortin-1-receptor (MC1R) associated with coat color (Edea et al., 2020; Sweet-Jones et al., 2021), and an essential regulator in the immune system factor regulatory factor X-5 (RFX5; Zhao et al., 2017; Chen et al., 2020). These genes may be related to the milk production traits, fur color, and immune response of Subei yaks.

## Conclusions

In this study, SNP markers detected by whole-genome resequencing technology were used. Although there was obvious gene flow between Subei yak, other domestic yak and wild yak, through population stratification analysis, it was proved that Subei yak population could be distinguished from other domestic yak populations and wild yak populations in China. The selective clearance analysis of Subei yak and other domestic yaks showed that the positive selection genes in Subei yak were mainly enriched in auditory receptor cell development, inositol phosphate metabolism, synthesis, and degradation of ketone bodies. These results suggest that Subei yaks may exhibit unique breed characteristics in terms of external environment perception, hypoxia adaptation, and meat quality. In summary, the study provides a reference for analyzing the population structure and elite germplasm characteristics of Subei yak. Furthermore, a theoretical basis for the resource conservation and utilization of Subei yak and subsequent variety improvement is also provided.

## Supplementary Material

skae152_suppl_Supplementary_Materials

## Abbreviations

CNV: copy number variation
DO-YAK: domestic yak
FST: fixation statistics
GN-YAK: gannan yak
GY-YAK: gaoyuan yak
GS-YAK: gaoshan yak
GO: gene ontology
HH-YAK: huanhu yak
JC-YAK: jinchuan Yak
JL-YAK: jiulong Yak
KEGG: kyoto encyclopedia of genes and genomes
NJ: neighbor-joining
PCA: principal component analysis
SVs: structural variants
SB-YAK: subei yaks
TZB-YAK: tianzhu white yak
