# Supplementary figures and images for: Whole-genome resequencing reveals the uniqueness of Subei yak
Source: J Anim Sci. 2024 Jun 4;102:skae152. doi: 10.1093/jas/skae152 (PMC11217902; doi:10.1093/jas/skae152)

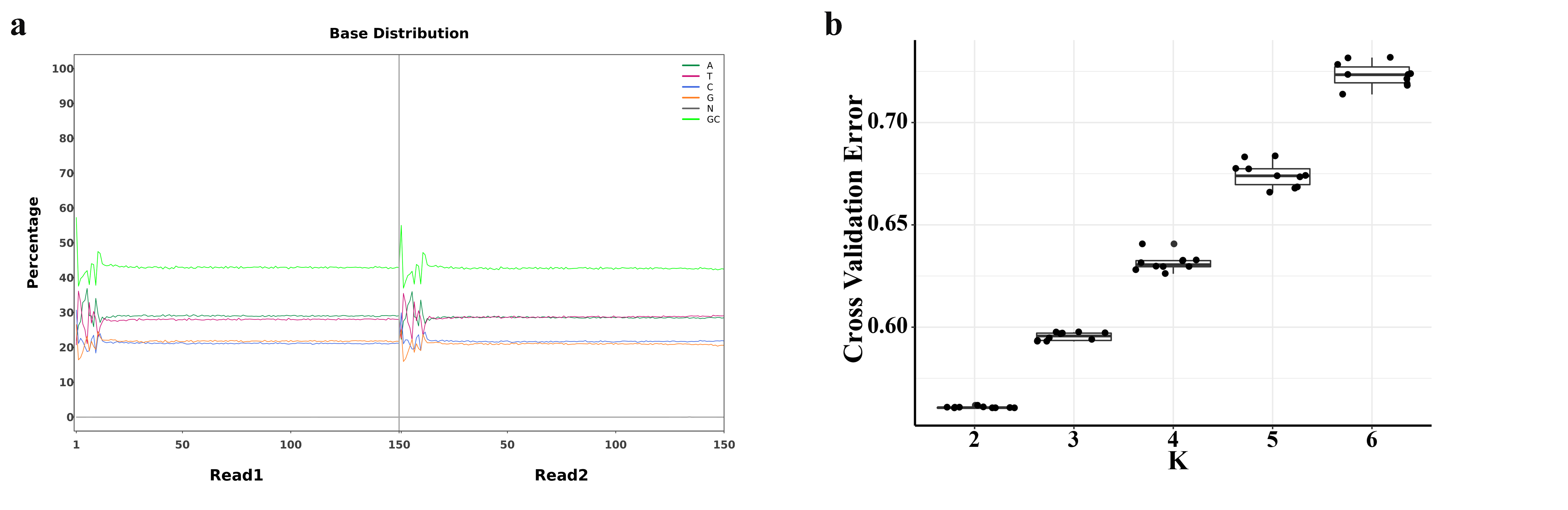

Supplement: skae152_suppl_Supplementary_Materials [file skae152_suppl_supplementary_materials.zip › Supplementary Figure 1.tif]
